# Editorial: Combined artificial intelligence and molecular dynamics (AI-MD) methods

**DOI:** 10.3389/fmolb.2022.1012785

**Published:** 2022-10-06

**Authors:** Leili Zhang, Kai Huang, Yi Isaac Yang, Liang Shi

**Affiliations:** ^1^ IBM Thomas J Watson Research Center, Yorktown Heights, NY, United States; ^2^ Shenzhen Bay Laboratory, Shenzhen, China; ^3^ University of California, Merced, Merced, CA, United States

**Keywords:** molecular dynamics simulation, artificial intelligence, protein folding, coarse-grained (CG) methods, quantum chemistry

After decades of theoretical and computational developments, Molecular dynamics (MD) simulation has not only become a tool that complements experimental interpretations and predictions, but it is also the benchmark for higher-level simulations. When we consider each of the components required for MD simulations: the theoretical engine (for example, Newton’s Laws of Motion, Laws of Thermodynamics, Langevin equation, Poisson–Boltzmann equation, etc.), the force field (parameters to calculate potential energy and forces), the propagation algorithm (for example, Verlet integration), the system (typically consisting of coordinates, velocities and connections), the controlling parameters (such as temperature, pressure, etc.), and the observables (for example, free energy calculations, collective variable monitoring, etc.), each of the components has come a long way from careful manual discoveries, designs, tunings and deployments. MD simulation has seemingly “matured” in most aspects, achieving roughly ∼1 kcal/mol of accuracy for binding free energy calculations with biological systems. However, problems such as folded/unfolded protein structure sampling, protein-protein interaction structure sampling, large system simulations, rare event simulations, simulations with non-negligible nuclear quantum effects, reactions, parameterization of new materials, and high-throughput free energy calculations persist, calling for new research and developments. Naturally, one could turn to Artificial Intelligence (AI), which is another field that has been significantly accelerated by the silicon revolution. Needless to say, AI has shown its usefulness in MD-related fields, especially in this “post-AlphaFold era”. Now the question is not an “IF”, but HOW we combine these two powerful tools to further both fields of research.

## Combining AI and MD to generate system coordinates

As was demonstrated by AlphaFold, AI combined with MD-inspired metrics leads to accurately predicted folded states of proteins (global minima, as observed by typical crystallized experimental structures). In our research topic, Tian et al. showcased the effectiveness of an algorithm combining MD simulation, Markov state models (MSM) and autoencoders/variational autoencoders. It samples the excited states and unexplored conformations of proteins, with plausible physical properties. While the authors used a bimodal protein as an example, the method could be easily extended to intrinsically disordered proteins, helping researchers explore missed structural spaces.

## Combining AI and MD to enhance simulated observables

Accurately simulated observables arguably are the “holy grail” of all simulations. Collective variables (CV), such as bond lengths, bond angles, etc., are often used in free energy calculations as “stepstone observables” that allow the system to move either rationally or radically. More complicated CVs (typically the combinations of simple CVs) have been designed in the literature for a researched system to access otherwise hard-to-access structural spaces. In this topic, Spiwok et al. designed a novel CV (AlphaFold CV, derived from its namesake). Combining AlphaFold CV and parallel tempering metadynamics simulation allowed the authors to explore possible structures of Trp-cage and β hairpin in under 100 ns, saving hundreds of microseconds of simulations required by a regular MD. The authors cautioned that even though using AlphaFold CV may accelerate structural explorations, fast divergences and unphysical configurations are likely to be encountered in the simulations. Therefore, before a “more focused version of AlphaFold CV” has been developed, a complementary second CV is recommended.

## Combining AI and MD for the propagation algorithm

For multi-scale simulations, the propagation algorithm on each scale needs careful coupling so that the system will propagate physically. Zhang et al. have developed an online machine learning algorithm for a theoretical deformable platelet motion equation (learned Jeffery orbits equation, LJOE) that was shown to accelerate the simulations by 99% once the parameters have stabilized (because only numerical calculations from the equations are required thereafter). Intriguingly, the authors have shown that past theoretical models of platelet motions are special cases of LJOE, where the platelets were either rigid or under special flow conditions. Combined with the multiscale modeling engine the authors have devised, this online machine learning model can possibly push the envelope of simulations to real-life time scales in the future. Interestingly, the authors have also developed an “AI-enhanced adaptive time stepping algorithm” for their multiscale modeling engine (consisting of all-atom MD, coarse-grained MD and dissipated particle dynamics), which is relevant to this topic.

## Combining AI and MD for force field developments

Parameters of water have always been the center stage in force field developments for MD simulations. Wu et al. stressed the importance of nuclear quantum effects (NQE) in simulations that include water and showed that under the assumption of the locality of quantum force correction, AI-learned potential term can significantly improve the water radial distribution function over some of the best current water models, while retaining classical MD speed. The authors suggested a similar AI-learned term be used for more systems where NQE is pronounced, as long as the assumption of locality holds.

Within our limited presentation, the authors who have contributed to our topic show that AI has been applied in vastly different areas of MD simulations. We also discover a common theme among the races to apply AI to MD: faster, larger, and preciser. More intricately, not only have tailored MD data and data representations benefited the studies, but tailored algorithms are also used for specific tasks to achieve better results. Ultimately, both the designed data structures and algorithms are combined to push the boundaries of simulations.

